# Supplementary material for: Detailed Characterization of the Lung–Gut Microbiome Axis Reveals the Link between PD-L1 and the Microbiome in Non-Small-Cell Lung Cancer Patients
Source: Int J Mol Sci. 2024 Feb 15;25(4):2323. doi: 10.3390/ijms25042323 (PMC10889071; doi:10.3390/ijms25042323)
Supplement: Supplementary file 1 [file ijms-25-02323-s001.zip › Supplementary Tables and Figure Captions.pdf]

Supplementary Table S1. A permutational multivariate analysis of variance in microbiome composition between samples.

Supplementary Table S2. Differential analysis of bacterial abundance in lung tumor and lung parenchyma tissue.

Supplementary Table S3. Differential analysis of the stool microbiomes between the NSCLC patients and control group.

Supplementary Table S4. β-diversity analysis along the lung–gut axis.

Supplementary Table S5. β-diversity analysis between NSCLC patients' samples and smoking habits.

Supplementary Table S6. β-diversity analysis between NSCLC patients' samples and COPD.

Supplementary Table S7. β-diversity analysis between NSCLC patients' samples and histological type of lung cancer.

Supplementary Table S8. β-diversity analysis between NSCLC patients' samples and degree of histologic differentiation in cancer cells.

Supplementary Table S9. β-diversity analysis between NSCLC patients' samples and PD-L1 expression on non-small cell lung cancer tissue.

Supplementary Table S10. β-diversity analysis between NSCLC patients' samples and $CD8^{+}$ T cells in non-small cell lung cancer tissue.

Supplementary Table S11. β-diversity analysis between NSCLC patients' samples and lipopolysaccharide level.

Supplementary Table S12. β-diversity analysis between NSCLC patients' samples and stage of lung cancer.

Supplementary Figure S1. (**A**–**D**) Venn diagram of unique bacterial sequences of all detected ASV.

Supplementary Figure S2. (**A**–**D**) Venn diagram of unique bacterial sequences of bacterial composition at the genus level.
