# Supplementary figures and images for: Detailed Characterization of the Lung–Gut Microbiome Axis Reveals the Link between PD-L1 and the Microbiome in Non-Small-Cell Lung Cancer Patients
Source: Int J Mol Sci. 2024 Feb 15;25(4):2323. doi: 10.3390/ijms25042323 (PMC10889071; doi:10.3390/ijms25042323)

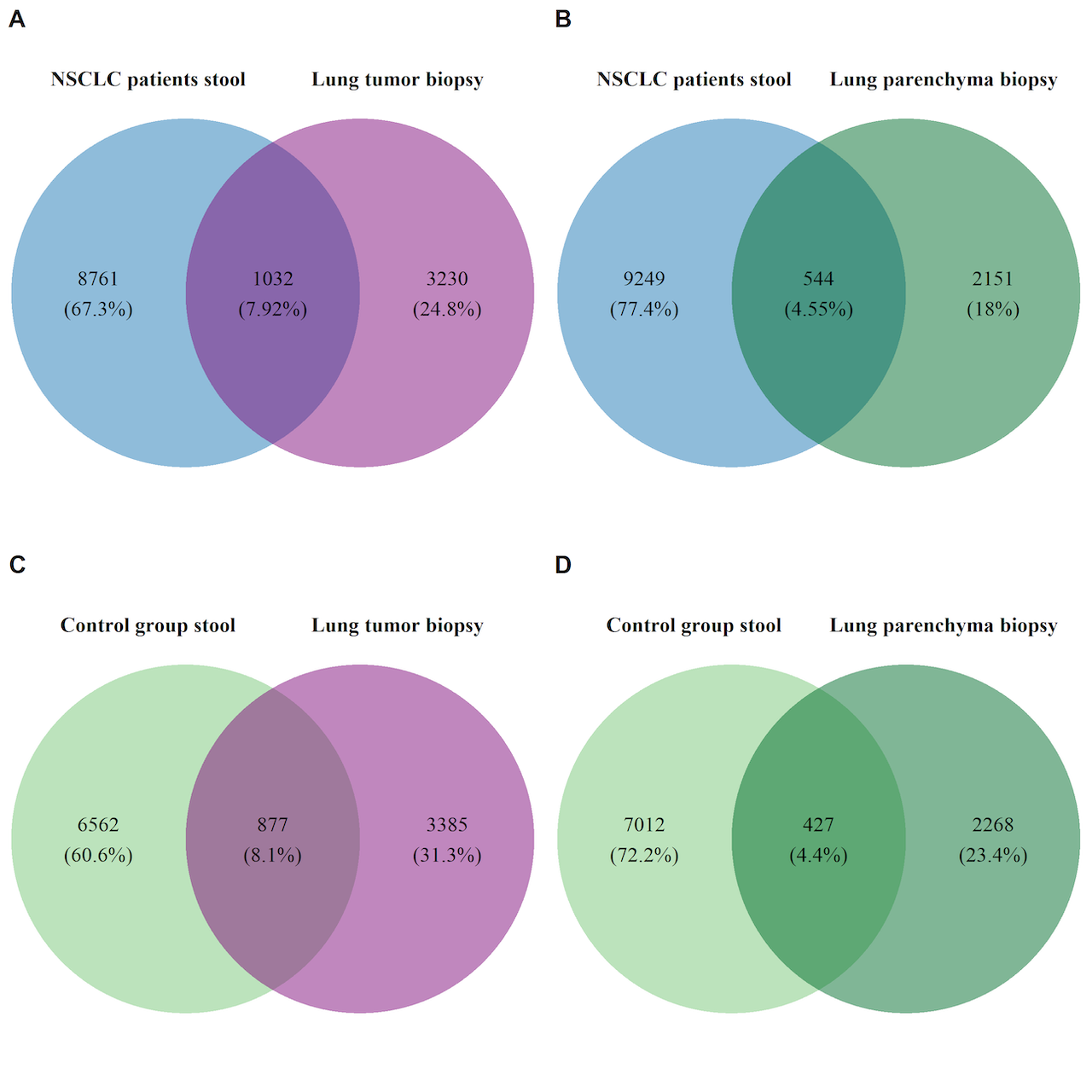

Supplement: Supplementary file 1 [file ijms-25-02323-s001.zip › Supplementary Figure S1. Venn Stool vs biopsy ASV 1.tiff]

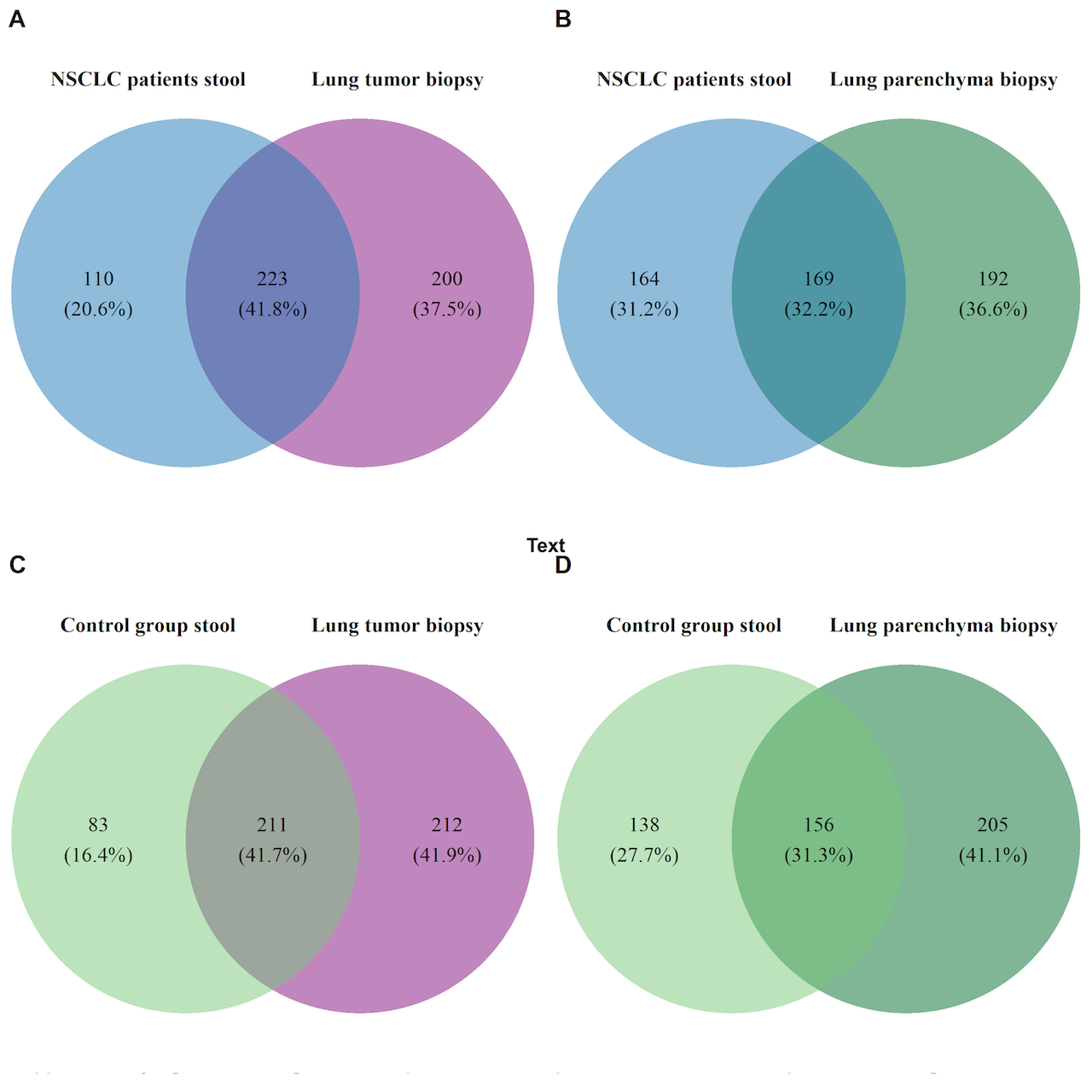

Supplement: Supplementary file 1 [file ijms-25-02323-s001.zip › Supplementary Figure S2. Venn Stool vs biopsy GENUS 1.tiff]
